# Potential Degradation of Swainsonine by Intracellular Enzymes of *Arthrobacter* sp. HW08

**DOI:** 10.3390/toxins5112161

**Published:** 2013-11-14

**Authors:** Yan Wang, Yanhong Li, Yanchun Hu, Jincheng Li, Guodong Yang, Danju Kang, Haili Li, Jianhua Wang

**Affiliations:** College of Veterinary Medicine, Northwest A & F University, Yangling, Shaanxi 712100, China; E-Mails: lyhdtiankong@126.com (Y.L.); hychun114@163.com (Y.H.); sdauvet2006@163.com (J.L.); ygd2002@126.com (G.Y.); kangdanju@126.com (D.K.); haili142@163.com (H.L.)

**Keywords:** swainsonine, *Arthrobacter* sp., mice, intracellular enzyme, degradation

## Abstract

Swainsonine (SW) is a toxin produced by locoweeds and harmful to the livestock industry. Degrading SW by *Arthrobacter* sp. HW08 was demonstrated as a promising way to deal with SW poisoning. However, it is unknown which part of the subcellular enzymes in *Arthrobacter* sp. HW08 is responsible for biodegrading SW and whether the metabolites are atoxic. In this study, intracellular and extracellular enzymes of *Arthrobacter* sp. HW08 were isolated and their enzyme activity was evaluated. The metabolites were fed to mice, and physiological and histological properties of the treated mice were investigated. The results showed that only intracellular enzyme of *Arthrobacter* sp. HW08 (IEHW08) could degrade SW efficiently. Compared with mice in SW treatment group, mice in SW + IEHW08 treatment group (1) increased their body weights; (2) showed higher number of platelets and lower number of white blood cells; (3) decreased the levels of creatinine, urea nitrogen, alanine transaminase and aspartate aminotransferase in serum; (4) reduced the number of vacuolated cells in cerebellum, liver and kidney. All these data demonstrate that IEHW08 was potentially safe for mice, while keeping the capacity of degrading SW. This study indicates a possible application of IEHW08 as an additive in the livestock industry to protect animals from SW poisoning.

## 1. Introduction

Swainsonine (SW) is a toxin produced by locoweeds and harmful to livestock industry [1,2]. Chronic ingestion of SW by livestock can cause many problems, including locoism, birth defects, reproductive disorders, congestive heart failure, edema growth retardation and loss of body condition [3,4,5]. 

To deal with SW poisoning, several methods have been attempted by managing locoweeds or livestock [4,6,7,8,9,10]. Biodegrading SW by bacteria was proposed firstly as a promising strategy to resolve this problem by our group [10,11]. *Arthrobacter* sp. HW08 was once isolated and characterized as potential bacteria to degrade SW in our previous study [11,12]. However, biological safety is a concern when the bacteria are fed to animals directly. Whether cell-free extract of *Arthrobacter* sp. HW08 remains the capacity of biodegrading SW is unknown. In this study, intracellular and extracellular enzymes of *Arthrobacter* sp. HW08 were purified and their capacity of degrading SW was compared by *in vitro* biochemical reaction. The metabolites of SW after degrading were fed to mice by intragastric administration. Biochemicals, physiological and histological properties of the treated mice were investigated. The results showed that intracellular enzymes of *Arthrobacter* sp. HW08 (IEHW08) were biologically safe for mice while keeping the capacity of degrading SW. This study suggests a possible application of IEHW08 as forage additives in livestock industry to prevent animals from locoweeds poisoning.

## 2. Results and Discussion

### 2.1. Degrading SW by IEHW08

**Figure 1:**
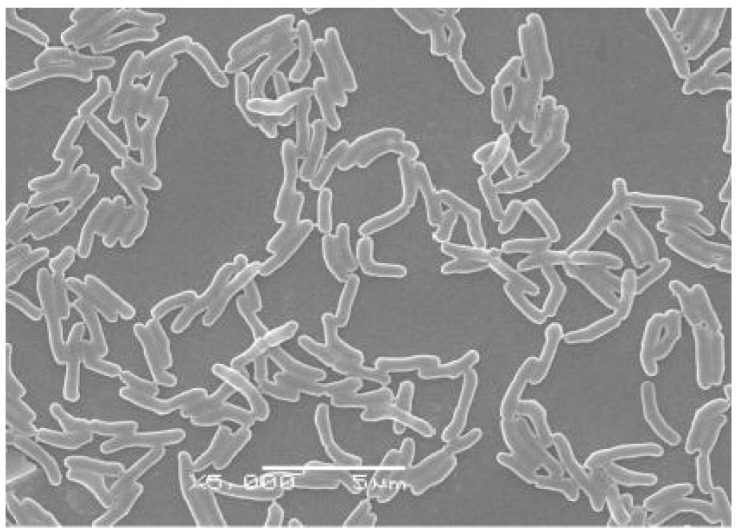
The morphology of HW08 bacteria observed by scanning electron microscopy. The bacteria are rod-like in shape. Scale bar = 5 μm.

**Figure 2:**
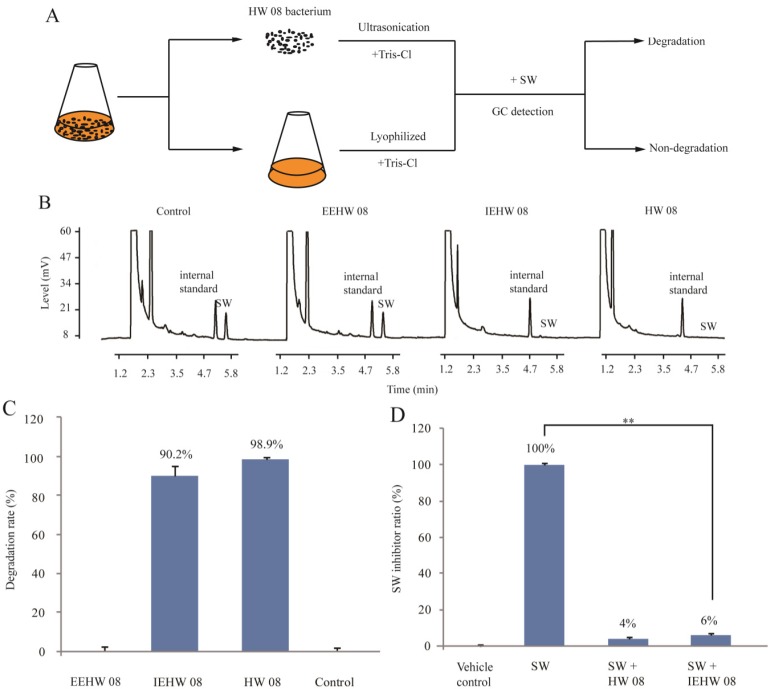
Degradation of SW by IEHW08 *in vitro*. (**A**) Flow diagram of isolating EEHW08 or IEHW08 for degrading SW; (**B**) GC analysis of residual SW after incubating SW with Tris-Cl (Control), EEHW08, IEHW08 and HW08, respectively; (**C**) SW degradation rate of EEHW08, IEHW08 and HW08. Tris-Cl was used as vehicle control; (**D**) Alfa-glycosidase inhibition rate of residual SW after incubating same dose of SW with IEHW08 and HW08, respectively. Tris-Cl was used as vehicle control while Tris-Cl plus SW as positive control.

*Arthrobacter* sp. HW08 was found to degrade SW in our previous study [11]. The morphology of growing HW08 bacteria was first observed by scanning electron microscopy. All the bacteria were rod-like in shape (Figure 1). To investigate which part of subcellular enzymes was responsible for degrading SW, we isolated EEHW08 and IEHW08, and further detected their ability of degrading SW *in vitro*. The whole procedure was illustrated in Figure 2A. The GC analysis showed that there were no residual SW that could be detectable after incubating SW with IEHW08 or HW08 (Figure 2B). These results indicate that IEHW08 could degrade SW as efficiently as HW08 did. In contrast, EEHW08 had no capacity of SW-degrading (Figure 2B). The SW-degrading rate of IEHW08 was also quantified (Figure 2C). SW is a specific inhibitor of alpha-mannosidase. To further confirm the SW-degrading capacity of IEHW08, a SW solution after reaction with IEHW08 *in vitro* was used to assay the inhibition rate of alpha-mannosidase. The result showed that the SW solution significantly lost the capacity of inhibiting alpha-mannosidase after reacting with IEHW08 *in vitro* (Figure 2D). All the data here demonstrate that IEHW08 is responsible for degrading SW.

### 2.2. Metabolites of SW Degraded by IEHW08 Was Safe to Mice

**Figure 3:**
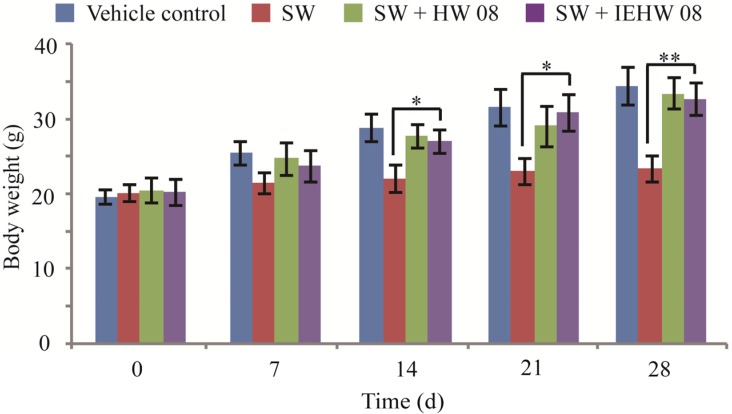
Body weight of mice in different treatment groups during the experiment period. Body weight of mice in SW + IEHW08 group is significantly higher than that in SW group after 14 days experimentation. * indicates *p* < 0.05; ** indicates *p* < 0.01.

**Figure 4:**
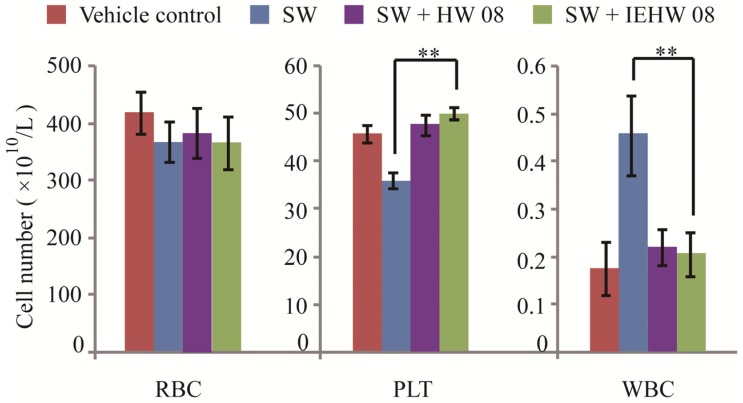
Comparison of blood routine examination indexes. Blood samples were collected from mice intragastrically administered by Tris-Cl (Vector control), Tris-Cl plus SW(SW group), and metabolites of SW degraded by IEHW08 or HW08 (SW + HW08 group, SW + IEHW08 group). The number of red blood cells (RBC), platelets (PLT) and white blood cells (WBC) was calculated and compared. ** indicates *p* < 0.01.

To investigate whether metabolites of SW degraded by IEHW08 were safe to animals, we did mice feeding experiments for 28 consecutive days. The records showed that mice in different groups exhibited significant differences in body weight after two weeks of experimentation. The body weight of mice in SW + IEHW08 group was significantly higher than that in the SW group (Figure 3). The similar case was observed in SW + HW08 group (Figure 3). Blood routine examination showed that there was significant difference between SW group and SW + IEHW08 group on the number of platelets and white blood cells. However, we did not find significant difference on the number of red blood cells among all treatments (Figure 4). 

**Figure 5:**
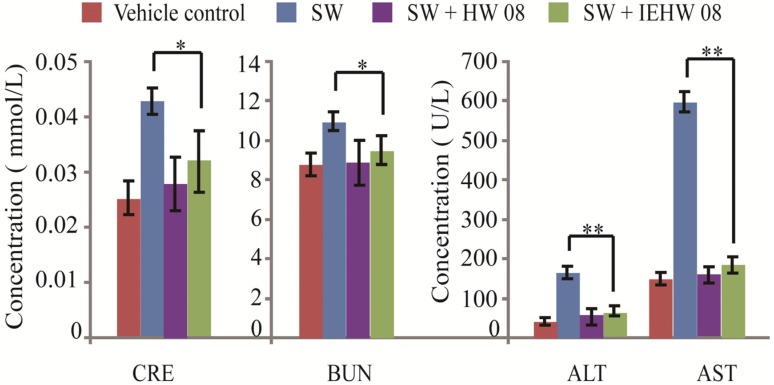
Comparison of blood physiological and biochemical indexes. Mice intragastrically administered with metabolites of SW degraded by IEHW08 and HW08 (SW + IEHW08 group, SW + HW08 group) showed significantly lower concentration of creatinine (CRE), blood urea nitrogen (BUN), alanine transaminase (ALT) and aspartate aminotransferase (AST) than that of mice intragastrically administered with swainsonine (SW group). Tris-Cl was used as vehicle control.* indicates *p* < 0.05; ** indicates *p* < 0.01.

Blood physiological and biochemical indexes were also compared by analyzing the blood samples from mice among different treatment groups. The results showed that concentrations of creatinine and urea nitrogen from mice in the SW + IEHW08 group were significantly lower than that in the SW group (Figure 5). In addition, the levels of alanine transaminase and aspartate aminotransferase from mice in the SW + IEHW08 group were significantly less when compared with that in the SW group (Figure 5). However, we did not find any significant differences among different treatment groups on the levels of triglyceride and alkaline phosphatase (data not shown). 

**Figure 6:**
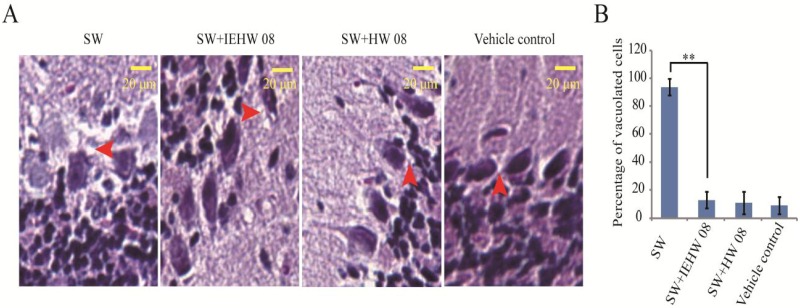
Histopathological evaluation of cerebellum. (**A**) Mice intragastrically administered with SW showed notable degenerative vacuolar changes in cerebellum. Arrow indicates vacuolation of Purkinje neurons. Mice intragastrically administered with metabolites of SW degraded by IEHW08 or HW08 had only a few degenerative vacuolar changes in Purkinje neurons. Mice intragastrically administered with Tris-Cl were used as vehicle control. Scale bar = 20 μm; (**B**) Quantification of vacuolated cells in each treatment. ** indicates *p* < 0.01.

**Figure 7:**
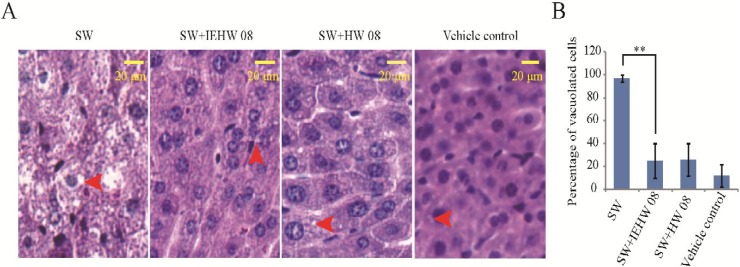
Histopathological evaluation of liver. (**A**) Mice intragastrically administered with SW showed evident vacuolar changes in liver. Arrow indicates vacuolation of hepatocytes. Mice intragastrically administered with metabolites of SW degraded by IEHW08 or HW08 had significantly decreased number of vacuoles in hepatocytes. Mice intragastrically administered with Tris-Cl were used as vehicle control. Scale bar = 20 μm; (**B**) Quantification of vacuolated cells in each treatment. ** indicates *p* < 0.01.

**Figure 8:**
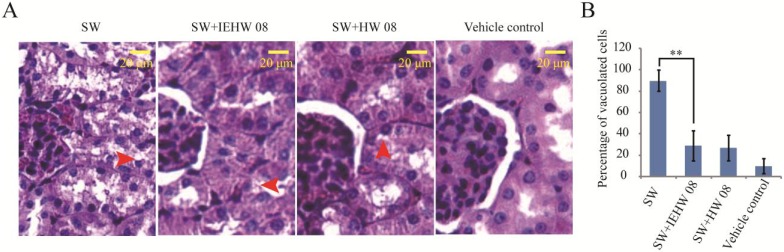
Histopathological evaluation of kidney. (**A**) Mice intragastrically administered with SW showed obvious vacuolar changes in renal corpuscle. Arrow indicates vacuolation of renal cells. Mice intragastrically administered with metabolites of SW degraded by IEHW08 or HW08 had only several degenerative vacuolar changes in renal cells. Mice intragastrically administered with Tris-Cl were used as vehicle control. Scale bar = 20 μm; (**B**) Quantification of vacuolated cells in each treatment. ** indicates *p* < 0.01.

To further confirm that the metabolites of SW degraded by IEHW08 were safe to mice, we performed a histopathological evaluation. The results showed that mice in the SW group displayed degenerative vacuolar changes in cerebellum (Figure 6A), liver (Figure 7A) and kidney (Figure 8A). In the sections of cerebellum from SW group, about 94% Purkinje neurons showed degenerative vacuolar changes. However, mice in SW + IEHW08 group showed only about 13% vacuolated Purkinje neurons in the cerebellum sections (Figure 6B). A similar percentage of vacuolated Purkinje neurons (11% and 9%) was also observed in the cerebellum sections from SW + HW08 group or vehicle control group (Figure 6B). In addition, we found that 97% hepatocytes of liver sections from SW group had degenerative vacuoles (Figure 7B). In contrast, only about 25% hepatocytes of liver sections from SW + IEHW08 group or SW + HW08 group had degenerative vacuoles (Figure 7B). Histopathological sections of kidney from SW group also showed that about 90% renal cells were vacuolated (Figure 8B). It is noteworthy that only about 29% vacuolated renal cells were found in the kidney sections from SW + IEHW08 group. The similar percentage (27%) of vacuolated renal cells was found in the kidney sections from SW + HW08 group (Figure 8B).

### 2.3. Discussion

The present study demonstrated that IEHW08 could degrade SW as efficiently as HW08 bacteria did. Importantly, metabolites of SW degraded by IEHW08 were safe to mice, which indicate a possible application of IEHW08 in coping with locoweeds toxicity of livestock as additives. 

The pathology of locoweeds poisoning has attributed to that SW in locoweeds inhibits cellular alpha-mannosidase and results in lysosomal accumulation of incompletely processed oligosaccharides as vacuoles [13,14]. Most livestock develop significant histologic lesions at dose of 0.4 mg SW kg^−1^ body weight in several weeks [15,16]. However, it’s challenging to use large animals for SW poisoning experiments regarding to cost and management. Mice as a model of locoweed poisoning have been used successfully to evaluate histopathological changes in liver, kidney and cerebellum although mice are relatively resistant to SW toxicity [17]. In our preliminary experiment, a dose effect of SW on mice was tested (data not shown) and 60 mg SW/kg body weight per day was determined, ensuring that the mice exhibited significant lesions of SW poisoning without severe death. 

Our mouse model of SW poisoning faithfully recapitulated the changes of blood cells that occurred on domestic animals [18,19]. It is important to note that mice in the SW + IEHW08 group showed an improvement of these indexes (Figure 4). Significant lesions in the kidney and liver have also been reported as characteristic of SW poisoning [14,20]. Our mice model with only SW treatment showed the poisoning of these two organs on blood physiological and biochemical indexes (Figure 5), which is consistent with histopathological impairment of kidney and liver (Figure 7A and Figure 8A). To be noted, mice fed with metabolite of the same dose of SW after degrading by IEHW08 showed rescue effect of these two organs (Figure 5, Figure 7B and Figure 8B). 

Other neurological signs of SW poisoning displayed by livestock include headshaking, nystagmus, opisthotonus, incoordination and ataxia [15,21]. These clinical signs have been attributed to the damage of cerebellar Purkinje neurons in form of cellular vacuolization [22]. Our mice model recapitulated this histopathological impairment with high percentage of vacuolated Purkinje neurons. In contrast, mice fed with metabolite of the same dose of SW after degrading by IEHW08 showed only a few vacuoles in Purkinje neurons (Figure 6). In all, the often damaged organs (liver, renal and cerebellum) in locoism were investigated here from biochemical to histopathological level. The evidences demonstrate that IEHW08 could efficiently degrade SW and the metabolites are safe.

Body weight is an important parameter used in determining the toxic effect of SW [23]. In agreement with previous studies on goats [24], our mice model also produced a significant decrease in body weight gain. However, mice fed with metabolite of the same dose of SW after degrading by IEHW08 grew well as vehicle control mice did. The data of body weights indicate that IEHW08 could make livestock refrain from growth retardation.

All the results indicate a possible application of IEHW08 as additives in livestock industry to prevent animals from SW poisoning. However, about the variance in physiological conditions between livestock and mice should be concerned and carefully examined when put IEHW08 into practice. In the future study, the defined components of IEHW08 need to be purified. The molecular mechanism of SW degradation by IEHW08 also needs to be investigated further.

## 3. Experimental Section

### 3.1. Preparation of HW08 Cell-Free Extract and SW

The *Arthrobacter* sp. HW08 bacteria were grown in 500 mL Erlenmeyer flasks containing 200 mL Luria-Bertani broth under the condition as described in our previous study for 24 h [11]. The growing HW08 bacteria were collected for morphological observation by scanning electron microscopy (JSM-6360LV, JEOL, Peabody, MA, USA) [10]. HW08 bacteria were harvested by centrifugation at 5000*g* for 10 min. The culture supernatant obtained after centrifugation was filtered through 0.22 μm filtration membrane and lyophilized as extracellular enzymes. The harvested HW08 bacteria were re-suspended in Tris-Cl buffer (pH 8.0) for sonication (Sonics-vibracell ultrasonic processor, Sonics & Materials Inc, Newtown, CT, USA) on ice, keeping sonifier output at 400 W with each stroke of 2 s with 1 s interval. The sonication time was 5 min in total. These extract was centrifuged at 10,000*g* for 30 min. The resulting supernatant was collected, filtered through 0.22 μm filtration membrane and used as an intracellular enzyme source. 

SW (1*S*,2*R*,8*R*,8a*R*)-1,2,3,5,6,7,8,8a-Octahydroindolizine-1,2,8-triol) was extracted from locoweed *Oxytropis ochrocephala Bunge* by methods described in previous study [25,26]. The final SW with purity above 97% was used for experiment. 

### 3.2. Enzyme Activity

The activity of extracellular enzymes of HW08 (EEHW08) and IEHW08 was determined by biochemical reaction in water bath at 30 °C for 50 min. The reaction mixture consisted of 40 μg SW in 140 μL Tris-Cl buffer and 60 μL enzyme solutions. After reaction, the residual SW was measured by gas chromatography (GC) with procedure as described in our previous study [11]. SW degradation rate was calculated by the following formula: degradation rate (%) = (SW_initial_ − SW_residual_)/SW_initial_ × 100%. The residual SW after biochemical reaction was also determined by alpha-mannosidase assay as our previous description [27].

### 3.3. Animal Experiment

Animal experiments were conducted according to guideline of Northwest A&F University for caring and using laboratory animals. Sixty-four Kunming strain mice were purchased from the Laboratorial Animals Center of Xi’an Jiao Tong University. The mice were 8–10 weeks old and weighed 19–21 g. The mice were quarantined and were allowed one week for acclimatization to the animal facility environment prior to experimentation. Sixty-four mice were then randomly separated into four groups: Vehicle control group, SW group, SW + HW08 group and SW + IEHW08 group. Mice in the vehicle control group were fed with Tris-Cl. Mice in the SW group were fed with 60 mg SW/kg body weight per day by intragastric administration. The same dose (60 mg) of SW was firstly degraded by HW08 or IEHW08, and then the degradation supernatant *(i.e*., metabolites) was collected and lyophilized for mice feeding experiment, respectively. Mice in the SW + HW08 group were fed with lyophilized powder dissolved in Tris-Cl buffer after HW08 degrading. Mice in the SW + IEHW08 group were fed with lyophilized powder dissolved in Tris-Cl buffer after degrading by IEHW08. Each group consisted of eight female and eight male mice. The experiment was performed for 28 consecutive days. During this period, the mice were clinically evaluated and their body weights were recorded every week. 28 days after experimentation, blood samples were collected from the ocular venous plexus for blood routine examination. Serum was used for physiological and biochemical analysis. At the end of the experimentation, all the mice were killed and cerebellum, liver and kidney were collected for histopathological analysis. The specimens were fixed in 4% paraformaldehyde and embedded in paraffin. Haematoxylin and eosin (HE) staining of sections was routinely performed. For each organ, three representative sections were chose for quantification. Five random fields of each section were used for calculating vacuolated cells. 

### 3.4. Statistical Analysis

The significance of the differences among different treatment groups was determined using one-way analysis of variance followed by the Bonferroni post-hoc test using SPSS 13.0 software (Spss Inc., Chicago, IL, USA). The level of significance was set at a *p*-value of less than 0.05 or 0.01.

## 4. Conclusions

Here we demonstrate that IEHW08 is responsible for the degrading SW in bacteria HW08. IEHW08 could degrade SW efficiently as HW08 *in vitro*. The metabolites of SW degraded by IEHW08 were safe for mice, as evaluated by body weights, blood routine examination, blood physiological, and biochemical indexes and histopathological analysis. All the evidences indicate a potential application of IEHW08 as additives in livestock industry to prevent animals from SW poisoning in the future.
